# The impact of *bacillus pumilus* TS2 isolated from yaks on growth performance, gut microbial community, antioxidant activity, and cytokines related to immunity and inflammation in broilers

**DOI:** 10.3389/fvets.2024.1383262

**Published:** 2024-04-26

**Authors:** Chuangen Guo, Sirui Liu, Liangjiao Di, Shu Tang

**Affiliations:** ^1^College of Veterinary Medicine, Nanjing Agricultural University, Nanjing, China; ^2^Animal Disease Prevention and Control Center of Rongchang, Chongqing, China; ^3^Key Laboratory of Fertility Preservation and Maintenance of Ministry of Education, School of Basic Medical Sciences, Ningxia Medical University, Yinchuan, China

**Keywords:** *Bacillus pumilus*, yaks, broilers, inflammation, gut microbial community

## Abstract

Intensive poultry farming faces challenges like gut inflammation in the absence of antibiotics, resulting in reduced productivity, heightened susceptibility to enteric diseases, and other complications. Alternative strategies are needed to manage inflammation and maintain sustainable poultry production. Yaks living in high-altitude hypoxic environments have specialized gut microbes. However, yak probiotics remain largely uncharacterized. We previously isolated a strain of *Bacillus pumilus* (named TS2) from yaks and demonstrated its potential as a probiotic *in vitro*. Therefore, in this study, we evaluated the *in vivo* growth-promoting, antioxidant, immune, and anti-inflammatory effects of *Bacillus pumilus* isolated from yaks in broilers. We demonstrated the safety of TS2 isolated from yaks in broilers. Furthermore, we found that TS2 increased the average daily weight gain (ADWG) and reduced the feed conversion ratio (FCR). Supplementation with TS2 also improved the mucosal morphology, the ratio of villi to crypt cells, and enzyme activity. High-throughput sequencing showed that the abundance of *Lactobacillus* was higher in the TS2 treated broilers. Importantly, the serum level of malondialdehyde (MDA) was reduced and the levels of total antioxidant capacity (T-AOC) and superoxide dismutase (SOD) activity were increased in the low-dose TS2 group, while the inflammatory factors interleukin-1β (IL-1β), interleukin-6 (IL-6) and tumor necrosis factor-α (TNF-α) were downregulated compared with the control group. We demonstrated that TS2 supplementation can increase the overall growth performance and ameliorate the blood parameters related to inflammation and immunity in broilers.

## Introduction

The rapid expansion of intensive farming worldwide has led to increasing challenges in poultry production, such as multiple causes of gut inflammation. Factors associated with these farming practices, such as increased exposure to pathogens, higher animal density, poor quality of feed ingredients, changes in feed formulation, and heat stress, raise the risks of subclinical and/or clinical gut inflammation (1, 2). Exposure to these triggers for an extended period of time (days to weeks) can result in a chronic intestinal inflammatory state with serious consequences for poultry performance and health, as it can alter intestinal architecture and decrease digestibility. These changes can result in the disruption of digestive function, induction of a constant state of oxidative stress, and poor immune competence (3–7).

In the past, large quantities of antibiotics were used as feed additives to promote growth performance (8). However, many studies have confirmed that the misuse of antibiotics may lead to drug resistance in bacteria, cause antibiotic-associated diarrhea, and intestinal microbial imbalance (9). Therefore, the use of antibiotics as feed additives is now banned in many countries. Consequently, there is an urgent need for new growth promoters as replacements for antibiotics in intensive poultry farming to safeguard the intestinal health and promote chicken growth and development. Increasing evidence indicates that probiotics supplementation could promote animal growth and inhibit the pathogenic microorganism (10, 11). Probiotics are live microorganisms that can benefit a host by colonizing the intestines and producing antibiotic substances. Such beneficial microorganisms compete with harmful bacteria for nutrients, adhesion, and colonization in the intestinal mucosa, thereby reducing the stress response, enhancing immunity, and regulating the binding of cytokines to receptors to modulate immune responses (12). Probiotics have shown a variety of health benefits in animals. Some researchers have reported that dietary supplementation with *Clostridium butyricum* or *Bacillus subtilis* as alternatives for antibiotics promote growth performance, improve immune function and anti-oxidative status, and benefit the cecal microflora (11, 13, 14). Yaks can survive under hypoxic and extreme weather conditions and may therefore have specialized intestinal bacteria compared to animals that live on the plains at lower altitudes (15).

In previous studies, we isolated a strain of *Bacillus pumilus* (named TS2) from yaks and demonstrated its potential as a probiotic *in vitro*. Therefore, in this study, we assessed the effects of TS2 on growth performance, intestinal digestive capacity, and microbes as well as antioxidant capacity, and immune indices in broilers.

## Materials and methods

### TS2 culture

In our previous work, TS2 was isolated from yak feces, genetically identified, and selected for its beneficial properties, including the inhibition of common pathogens (e.g., *S. aureus*, *Salmonella*, and *E. coli*), and its tolerance to high temperature, high bile salts, and acidic environments. The strain TS2 was preserved in the China Center for Type Culture Collection with the number M2023246. TS2 was activated by incubating on Luria–Bertani (LB) agar at 37°C for 24 h. Then, a single colony was selected and incubated in LB broth (pH 7.0) for 24 h. After centrifugation of the bacterial cultures at 3,000 rpm at 4°C for 10 min, the supernatants were discarded. PBS diluent was then added to the bacterial pellet, and serial dilutions of the bacterial suspension (10^-3^–10^-7^) were performed. LB agar plates were inoculated with serial dilutions of TS2 bacterial suspension, which were incubated at 37°C for 24 h prior to colony enumeration. Finally, an appropriate dilution factor was selected for diluting the TS2 bacterial pellet for feeding to broilers.

### Animal experiments and sample collection

All procedures contributing to this work complied with the ethical standards of the China Laboratory animal-Guideline for ethical review of animal welfare and were performed following the approval of the Animal Welfare Committee of Nanjing Agricultural University (permit number NJAU.No20220607120). Eighty white-feather broilers (aged 1 day) were obtained from the Nanjing Agricultural University Animal Experiment Center. After 5 days of acclimatization, the broilers, with an average body weight of 100 g per group, were randomly divided into four groups (*n* = 20/group). The chicks were not individually identified or separated by gender. Broilers in the control group were orally gavaged with 200 μL of physiological saline daily for 15 consecutive days. Broilers in the other three groups received TS2 at doses of 1 × 10^9^ CFU (high-dose group), 1 × 10^8^ CFU (intermediate-dose group), and 1 × 10^7^ CFU (low-dose group) in the same volume (200 μL) by oral gavage for the same duration. The broilers were raised under standard hygienic conditions (temperature: 28 ± 2°C, humidity: 55% ± 2%, light: 12 h) and were allowed free access to food and water. The composition and nutrient levels of the basal diets are shown in Table 1 (16, 17). The body weight and feed intake of the broilers were recorded daily for the duration of the experiment. On the 21st day, all chicks from each group were euthanized, and five samples of the jejunal stools were selected. The duodenum, jejunum, and ileum of all euthanized chicks were collected for hematoxylin and eosin (H&E) staining. Blood samples were also collected, placed statically in the centrifuge tube for 1 h, and then centrifuged at 3,500 × g for 10 min at 4°C in a high-speed centrifuge. The supernatants were stored at − 20°C for ELISA and biochemical analysis.

**Table 1:** Composition and nutrient level of the basal diet^1^.

Item	5 ∼ 21 days
Diet composition (%)	
Corn	57.77
Soybean meal	29.24
Cotton seed meal	4.86
Fishmeal	2.43
Soybean oil	2.02
CaHCO_3_	1.54
Limestone	1.2
NaCl	0.35
Premix	0.25
Choline	0.18
Methionine	0.16
Nutrient levels	
Metabolizable Energy (MJ kg^−1^)	12.32
Crude protein	21.23
Lysine	1.10
Methionine+ Cystine	0.85
Calcium	1.01
Total phosphorus	0.42

^1^Supplied per kg of diet: Fe 80 mg, Zn 80 mg, Mn 70 mg, iodine 0.35 mg, Se 0.23 mg, VA 1.65 mg, VD3 0.18 mg, VE 20 mg, Vk 0.50 mg, VB12 1.2 mg, pantothenic acid 10 mg, pyridoxine 4.5 mg, niacin 35 mg, biotin 0.15 mg, riboflavin 3.0 mg, folic acid 0.45 mg, VA, vitamin A; VD3, vitamin D3; VE, vitamin E; VK, vitamin K; VB12, vitamin B12.

### Determination of digestive enzyme activity in the jejunum

Two grams of preprocessed jejunal contents were weighed and combined with 18 mL of PBS (pH 7.4) to form a 10% homogenate. Subsequently, the samples underwent centrifugation at 3000 g for 20 min at 4°C, following which the supernatant was extracted. The supernatant was then gradually diluted before being mixed with specific reagents capable of reacting with digestive enzymes, such as starch substrates, etc. After adding suitable substrates, the mixtures were incubated at 37°C for a set period. Reactions were terminated using a stop solution. The activities of amylase, lipase, and trypsin were assessed in accordance with the manufacturer’s instructions (Nanjing Jiancheng Bioengineering Institute, Nanjing, China). TECAN (Infinite 200 PRO series) microplate reader (Tecan, Mannedorf, Switzerland) was employed to determine the optical density (OD) values after the reaction.

### Histopathological examination

Samples of broiler intestinal tissue fixed in 4% paraformaldehyde were serially dehydrated using alcohol, clarified in xylene, and embedded in paraffin. The fixed samples were sectioned (4 μm thickness), stained with H&E and examined under a light microscope (Carl Zeiss, Gottingen, Germany). The length of small intestinal villi was measured by drawing a vertical line from the base to the top. Similarly, the depth of intestinal crypts was measured vertically from the bottom of the crypt to the Lamina propria of the small intestine. For analysis, three fields were chosen from each slice. Ten intestinal villi and intestinal crypts were sampled from each field and then measured and analyzed using the 3DHISTECH SlideViewer software (3DHistech Ltd., Budapest, Hungary).

### DNA extraction and high-throughput sequencing analysis

Approximately 0.2 g of intestine tissue was sampled from each sample for DNA extraction using a TaKaRa MiniBEST Bacteria Genomic DNA Extraction Kit (Takara Bio, Beijing, China). The concentration and purity of the DNA samples were determined using agarose gel electrophoresis. Genomic DNA (gDNA) concentration was quantified using a NanoDropTM spectrophotometer (Thermo Scientific, MA, USA), and the samples were diluted to 1 ng/μL with sterile water. The V3–V4 regions of the 16S rRNA gene were amplified by PCR with the primer pair ACTCCTACGGGAGGCAGCA (338F) and GGACTACHVGGGTWTCTAAT (806R) under the following conditions: 30 cycles of 98°C (15 s), 55°C (30 s), 72°C (30 s), followed by a final extension at 72°C for 5 min. PCR products were detected by electrophoresis in a 2% agarose gel containing ethidium bromide. The DNA library was constructed using a library building kit. After quantification and testing by Qubit, the library was sequenced using NovaSeq6000, with sequencing performed by Wuhan Feisha Genetic Information Co., Ltd.

Raw data underwent quality control, including removal of low-quality reads and length-based filtration, to generate high-quality reads. These reads were then clustered into operational taxonomic units (OTUs) or amplicon sequence variants (ASVs), referred to as features. Taxonomic annotation of feature sequences was performed using a Bayesian classifier with the SILVA ribosomal RNA database as a reference (SILVA). Composition statistics were calculated for each sample at the phylum, class, order, family, genus, and species levels. Abundance of each species in samples was obtained using QIIME program (Version 1.9.1), and distribution histograms at each taxonomic level were generated using R software (Version 4.2.0).

### Antioxidant-related and inflammatory parameters analysis

The antioxidant-related and inflammatory parameters, including interleukin-1β (IL-1β), interleukin-6 (IL-6), tumor necrosis factor-α (TNF-α), total antioxidant capacity (T-AOC), superoxide dismutase (SOD) activity, and malondialdehyde (MDA), were assessed using commercial assay kits (Nanjing Jiancheng Bioengineering Institute, Nanjing, China). The measurements were conducted strictly following the manufacturer’s instructions. Following the manufacturer’s instructions, various reagents were added as per the protocol, and the OD values after the reaction were determined using the TECAN (Infinite 200 PRO series) microplate reader (Tecan, Mannedorf, Switzerland).

### Statistical analysis

Statistical analysis was conducted using GraphPad Prism 9.0.0 software. Data were represented as the mean ± standard deviation (SD). Differences were compared using one-way analysis of variance (ANOVA). *p* < 0.05 was considered statistically significant, and *p* < 0.01 was considered to indicate a high degree of significance.

## Results

### Growth performance analysis

The effects of dietary supplementation with TS2 on the growth performance of broilers are shown in Table 2. At day 21, the average daily weight gain (ADWG) of broilers in the low-dose group was nearly 1.2-fold higher than the control group, 1.14-fold higher than the intermediate-dose group and 1.27-fold higher than the high-dose group (*p* < 0.05). The feed conversion ratio (FCR) was also markedly decreased in the low-dose group compared with that of the control group at this time point. These results indicate that TS2 supplementation improved the growth performance of broilers.

**Table 2:** Effects of TS2 on broilers growth performance.

Parameter	Control	Low	Intermediate	High	p
Days 5–21					
ADFI, g/day	72.01	80.12	75.30	70.02	
ADWG, g/day	46.95 ± 2.81^bc^	56.07 ± 3.02^a^	49.11 ± 1.23^ab^	44.3 ± 2.49^c^	<0.001
FCR	1.54 ± 0.09^ab^	1.43 ± 0.08^a^	1.53 ± 0.04^ab^	1.58 ± 0.09^b^	0.004

^a,b,c^Means with a different superscript in the same row differ at *p* < 0.05.

### Morphology of the small intestinal mucosa

**Figure 1:**
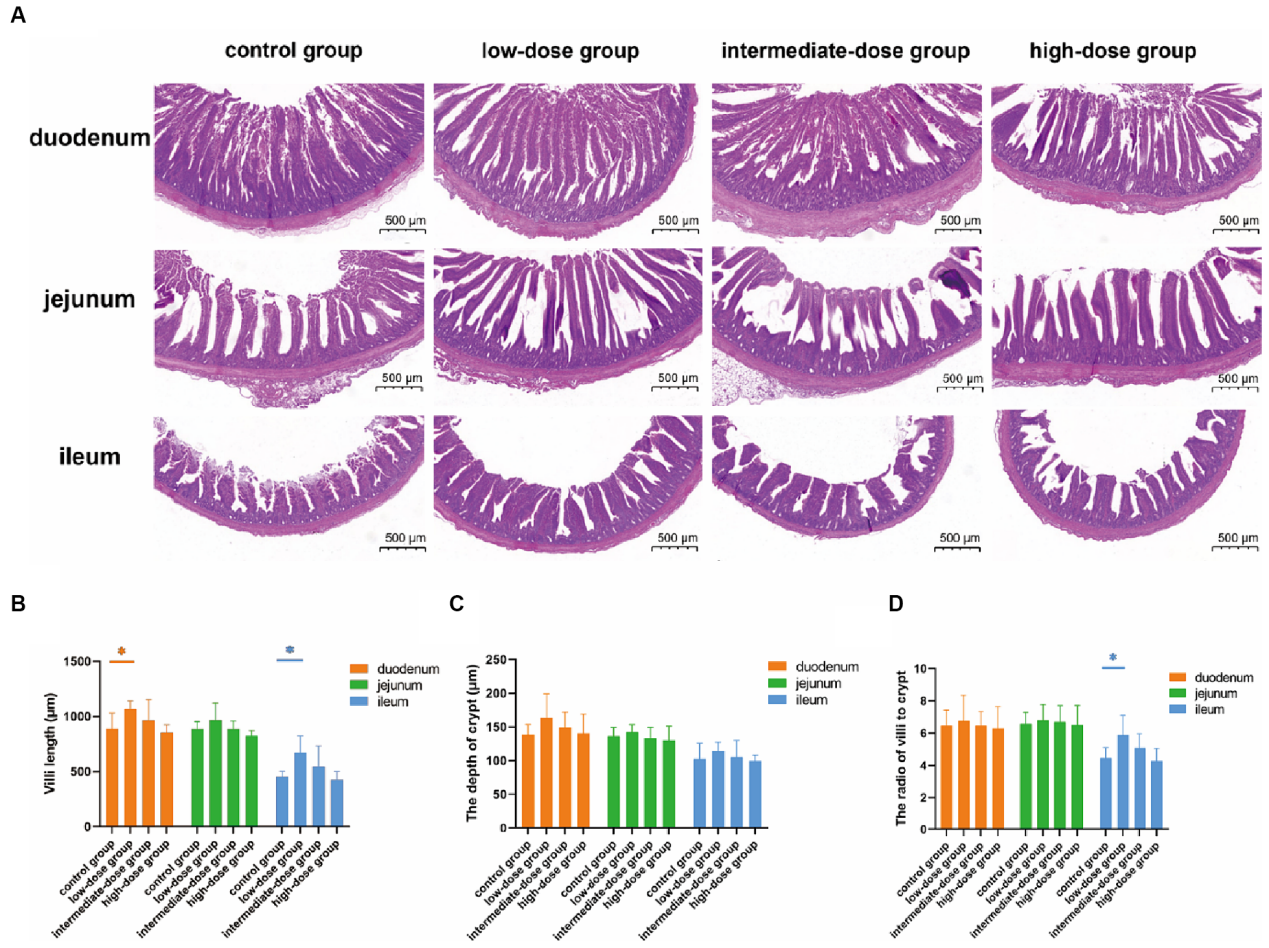
**(A)** H&E-stained histopathology of duodenum, jejunum, ileum. **(B)** Villus length and **(C)** crypt depth in the duodenum, jejunum, ileum. **(D)** Ratio of villus length to crypt depth. Data represent the mean ± SD. *p < 0.05, **p < 0.01.

The morphology of the intestines was analyzed after H&E staining (Figure 1A). Compared with the control group, the villi in the duodenum, jejunum, and ileum were elongated in the low-dose group, although the differences were statistically significant only in the duodenum and ileum (*p* < 0.05). Additionally, although not statistically significant, the lengths of villi in the duodenum, jejunum, and ileum of broilers in the intermediate-dose group showed an increase compared to the control group (Figure 1B). The crypt depth in the duodenum was also elongated in the low-dose group compared with that in the control group, although the difference was not statistically significant. However, there were no significant differences in the crypt depths among the control group and other dosage groups (Figure 1C). In addition, the villus length/crypt depth (V/C) ration in the duodenum, jejunum, and ileum were increased in the low-dose group compared with that in the control group, although the difference was statistically significant only in the ileum (*p* < 0.05; Figure 1D). These data indicate that low-dose TS2 supplementation significantly improved the morphology of the small intestinal mucosa.

### Activity of jejunal digestive enzymes

**Figure 2:**
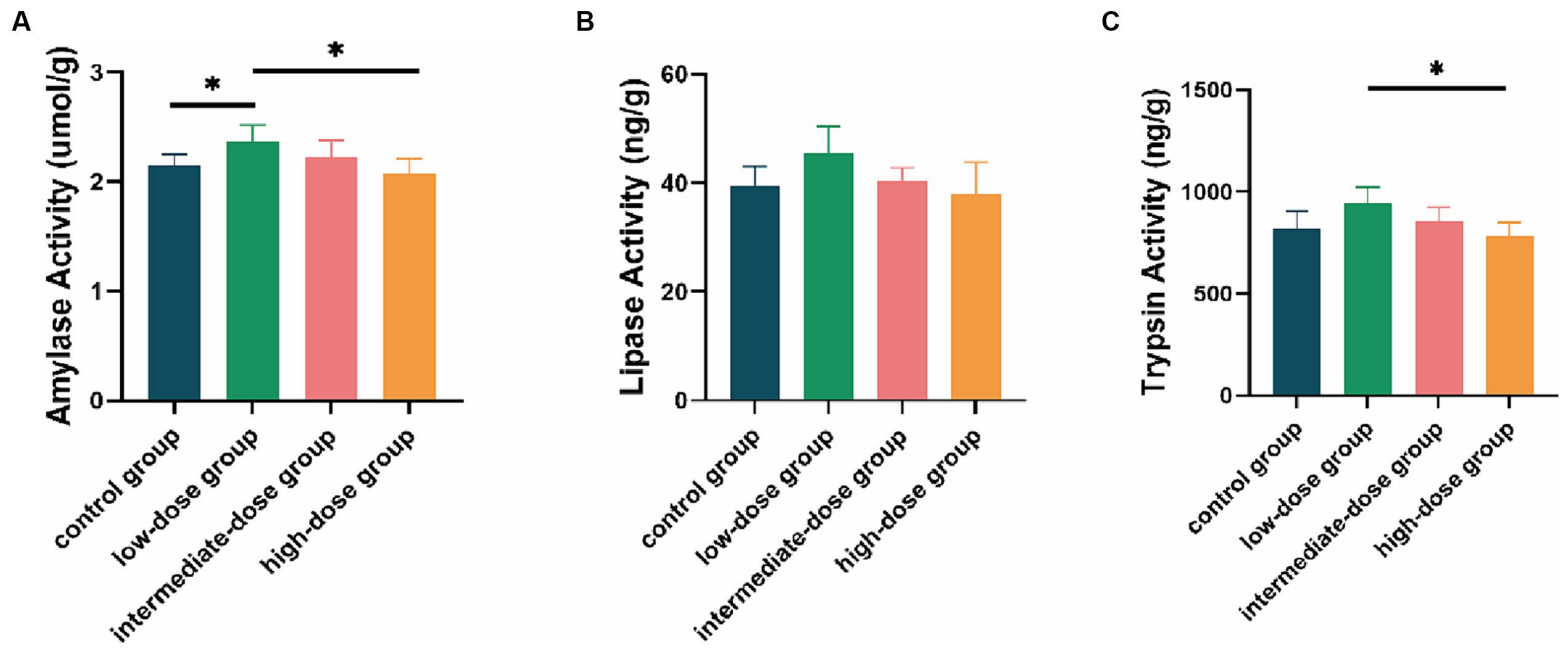
Enzyme activity in the intestinal tract of broilers. Effect of TS2 on jejunum **(A)** amylase, **(B)** lipase and **(C)** trypsin activities at 21 days. Data represent the mean ± SD. *p < 0.05, **p < 0.01.

At 21 days, compared with the control group and the high-dose group, the amylase activity in the intestinal tract of broilers in the low-dose group significantly increased (*p* < 0.05; Figure 2A). The lipase activity in the low-dose group was higher than that in the control group and other dosage groups (Figure 2B). Additionally, the trypsin activity in the low-dose group significantly increased compared to the high-dose group (*p* < 0.05) and was higher than that in the control group and other dosage groups (Figure 2C).

### Diversity of the microbial community in the jejunum

**Figure 3:**
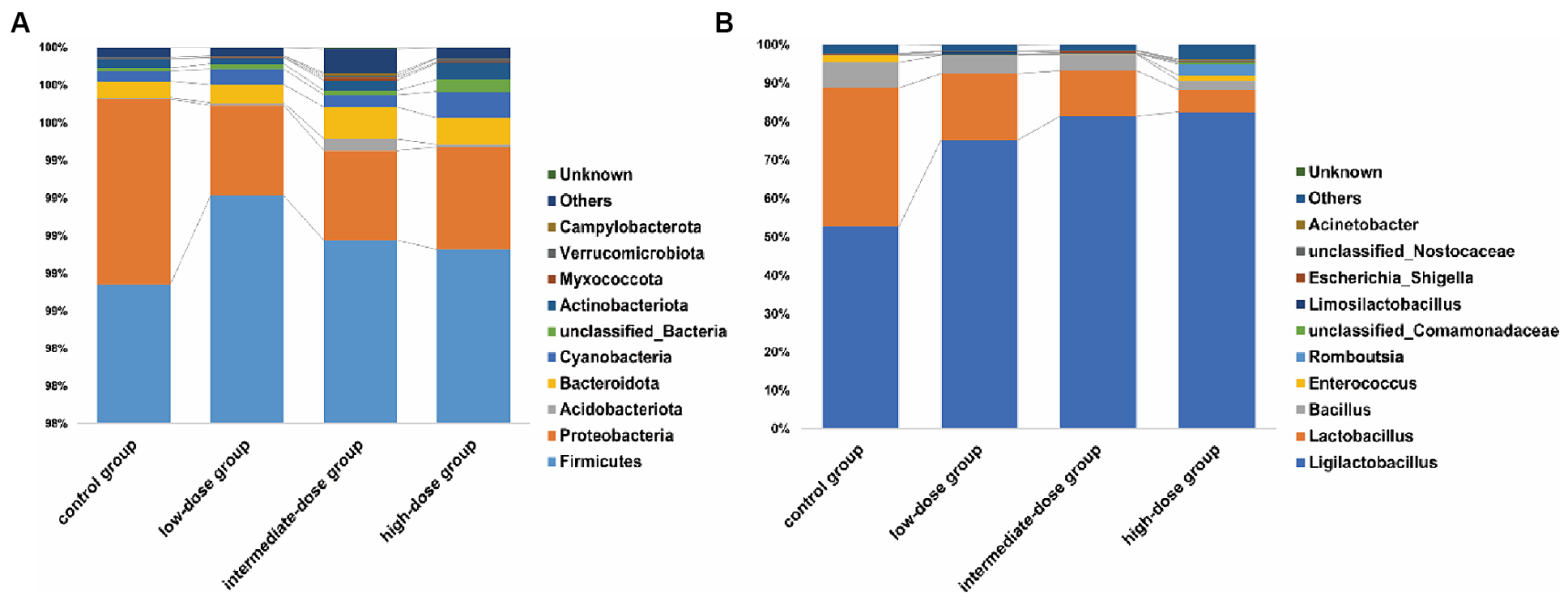
The main microbial community structure on day 21. **(A)** The main microbial community structure at the level of phylum in different samples. **(B)** The main microbial community structure at the level of genus in different samples on day 21.

A total of 3,121,585 pair end (PE) reads were generated by high-throughput sequencing of all samples. After PE read quality control and assembly, 3,115,714 clean reads were obtained. A minimum of 79,202 and an average of 79,890 clean reads were generated for each sample. The length distribution of the optimized sequence main was 400 bp–500 bp. The microbial communities in the jejunum were identified at different levels (Figure 3). At the phylum level (Figure 3A), Firmicutes and Proteobacteria predominated in the jejunum in the control and TS2-treated groups on day 21, although the percentage of Proteobacteria was lower in TS2-treated groups than that in the control group. At the genus level, the proportion of *Lactobacillus* was significantly higher in the low-dose group compared to the control group (Figure 3B). However, the percentage of *Bacillus* in the low-dose group was significantly lower than that in the control group.

### Antioxidant-related parameters

**Figure 4:**
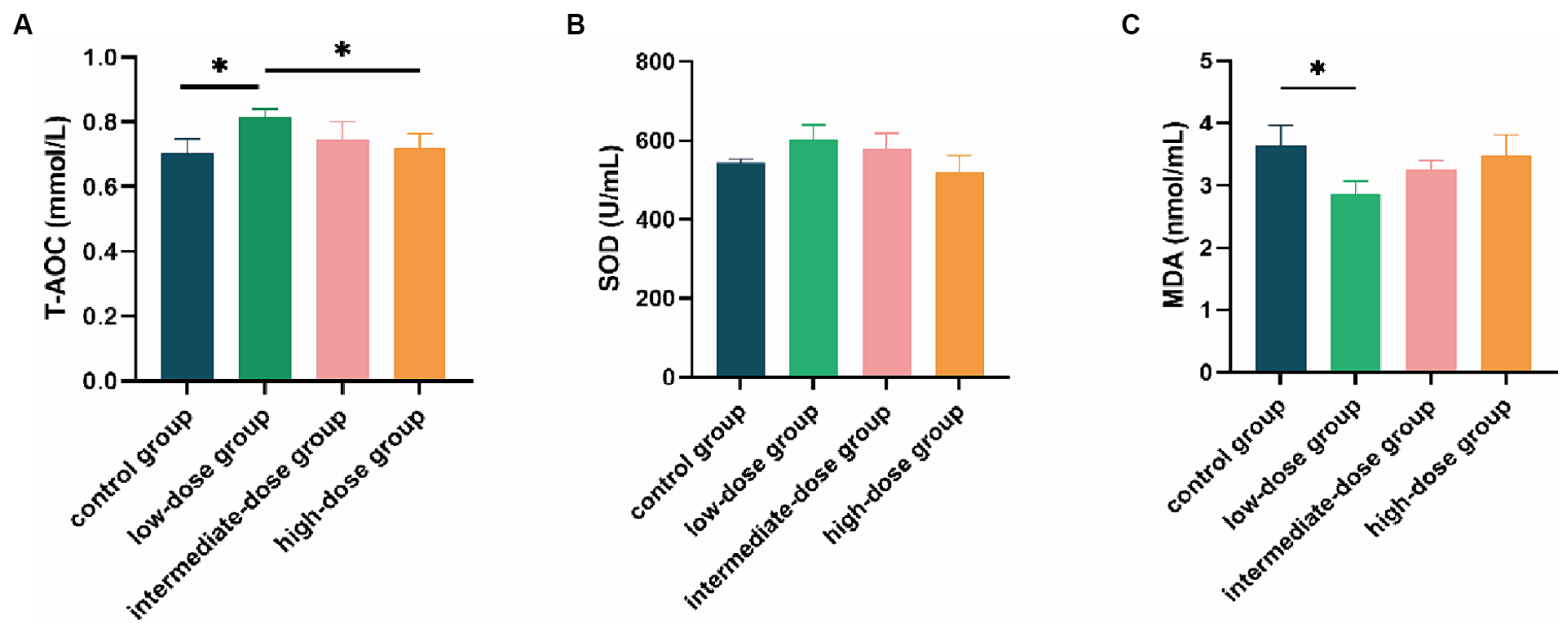
Effect of TS2 supplementation on various serum anti-oxidative parameters in broilers. **(A)** The T-AOC level of serum in each group. **(B)** The SOD of serum in each group. **(C)** The MDA of serum in each group. Data represent the mean ± SD. *p < 0.05, **p < 0.01.

Compared with the control group and the high-dose group, the serum T-AOC content in the low-dose group significantly increased (*p* < 0.05; Figure 4A). The SOD content in the low-dose group was also higher than that in the control group and other dosage groups (Figure 4B). In addition, the MDA content in the low-dose group was significantly lower than that in the control group (*p* < 0.05) and lower than that in other dosage groups as well (Figure 4C).

### Inflammatory factors

**Figure 5:**
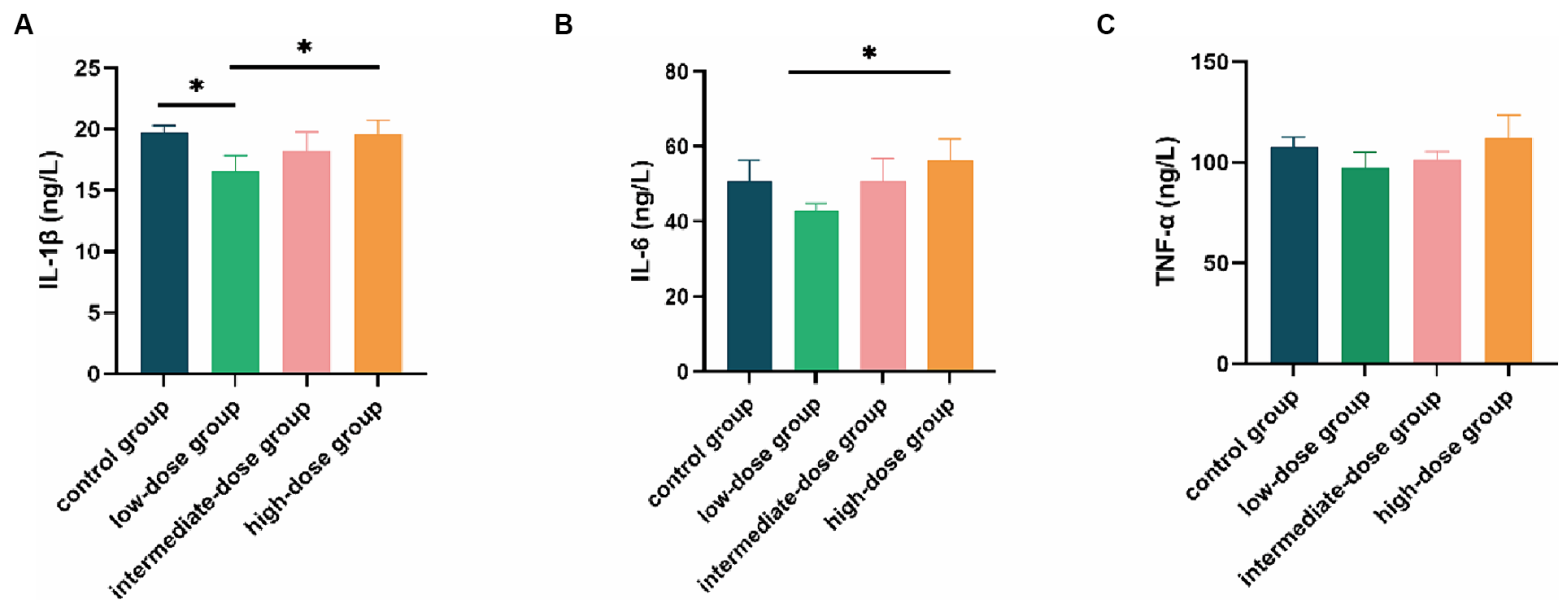
Effect of TS2 supplementation on various serum inflammatory factor in broilers. **(A)** Serum IL-1β, **(B)** IL-6, and **(C)** TNF-α level in the different groups. Data represent the mean ± SD. *p < 0.05, **p < 0.01.

In contrast to both the control and high-dose groups, the IL-1β content in the serum of broilers in the low-dose group was significantly reduced (*p* < 0.05; Figure 5A). Additionally, the IL-6 content in the low-dose group was significantly lower than that in the high-dose group (*p* < 0.05), and it was also lower than the control group and the intermediate-dose group (Figure 5B). Furthermore, the TNF-α content in the low-dose group was lower than that in the control group and other dosage groups (Figure 5C).

## Discussion

Intestinal health is crucial for maintaining general health and welfare, maximizing growth performance and feed efficiency, and mitigating the environmental impacts of both excessive undigested nutrients and the generated pollutants in broiler production, since this multifunctional organ is central to nutrient digestion and absorption, metabolism, immune response, and endocrine function (18, 19). Previous studies have shown that the bulk of nutrition absorption occurs in the small intestine and is positively correlated with the small intestinal villi length and crypt depth. The vast absorptive surface area provided by the increased intestinal villus length is considered to be a key factor in promoting growth performance (20). In the present study, our analysis of the morphology of the small intestine showed that the ratio of the small intestinal villi length compared with the depth of the crypt increased significantly when broilers received dietary supplementation with low-dose TS2, suggesting that low-dose TS2 supplementation significantly improved the growth of intestinal villi and depth of the crypt. This result consistent with previous research (11). Growth and development processes are critically dependent on the absorption of nutrients. In this research, the ADWG and FCR were significantly improved in the low-dose group on day 21. However, the ADFI in the intermediate and high-dose groups did not differ. Broilers supplemented with low-dose TS2 had a higher final body weight without increasing food intake, indicating improved absorption and digestion capabilities of the TS2-treated broilers. Thus, our results firstly indicate that dietary supplementation with low-dose TS2 can increase the length of intestinal villi and significantly improve the overall daily weight gain of broilers and it is safe to use in animals.

The intestinal bacterial community is a complex ecosystem consisting of a huge variety of interactions between microbes, which may have a significant impact on the host’s physiology (21, 22). At the phylum level, Firmicutes and Proteobacteria were the predominant bacteria in the jejunum of the control group and experimental groups. At the genus level, *Ligilactobacillus* was more abundant in the TS2-treated groups than in the control group. This may be because TS2 is more favorable for the growth of *Ligilactobacillus* colonization. In previous reports, *Lactobacillus salivarius* has frequently been isolated from chickens and demonstrated to possess antibacterial properties, while also showing potential in boosting chicken immunity (23, 24). Interestingly, we found a decrease in *Bacillus* in the TS2-treated groups, indicating that *Bacillus pumilus* exists temporarily in the broiler intestine. The effect of TS2 on body weight is likely to be achieved by providing the right nutrients and a favorable colonization environment for probiotics such as *Ligilactobacillus*. Low-dose TS2 has the capacity to enhance the proliferation of beneficial bacteria within the intestinal tract, notably *Ligilactobacillus*, thereby augmenting their presence and fostering a more balanced intestinal microecology. This increase in beneficial bacteria plays a pivotal role in upholding the integrity of the intestinal mucosa, curtailing the infiltration of detrimental substances, and mitigating the onset of inflammatory responses.

Oxidative stress occurs when the level of reactive oxygen species (ROS) and other oxidants exceeds the capacity of the antioxidant defense mechanisms, leading to cellular damage and dysfunction. T-AOC, SOD, and MDA are three major biomarkers that are closely related to oxidative stress (14, 25). T-AOC is a measure of the overall antioxidant defense system, reflecting the ability of antioxidants to neutralize ROS and other oxidants (26). Low T-AOC levels indicate a weakened antioxidant defense system and increased susceptibility to oxidative stress. As a primary antioxidant enzyme in cells, SOD plays a critical role in protecting cells against oxidative stress by scavenging superoxide radicals (27, 28). MDA is one of the end-products of membrane lipid peroxidation and its content can be used as an indicator to assess the severity of stress in cells (29). MDA has been shown to have detrimental effects on various cellular processes, including DNA damage, protein modification, and impairment of membrane function. In addition, MDA can induce the production of pro-inflammatory cytokines and activate various signaling pathways that promote cell death (30, 31). Environmental stress can accelerate the production of ROS and lead to oxidative stress (32). Oxidative stress can not only induce several diseases but also affect the growth rate, meat quality, and feed conversion rate of animals (33, 34). Earlier studies have shown that antioxidant enzymes can not only reduce ROS production and prevent oxidative stress, but also repair oxidant damage induced by oxidative stress (35, 36). Therefore, improving the activity of antioxidant enzymes is very important for the healthy growth and development of animals. Previous research has shown that probiotic supplementation can increase the levels of antioxidant enzymes, which are important for improving antioxidant capacity (37, 38). In this study, MDA levels were reduced and the levels of T-AOC and SOD were increased in the low-dose TS2 group, suggesting that TS2 supplementation boosts the antioxidant capacity of broilers. The antioxidant properties of low-dose TS2 enable it to scavenge free radicals within the body, thereby diminishing oxidative stress-induced damage to the intestinal mucosa. This preservation of intestinal mucosal integrity further contributes to the dampening of inflammatory responses.

IL-1β has strong pro-inflammatory activity and induces a variety of pro-inflammatory mediators, such as other cytokines and chemokines (39). IL-6 has been shown to regulate the activity of pathogenic T helper cells (Th cells) to amplify and perpetuate chronic inflammation (40). Furthermore, TNF-α expression is increased in mucosal tissue during inflammation (41). Accumulating evidence indicates that probiotic colonization in the intestines contributes to an anti-inflammatory environment and reduces the production of pro-inflammatory cytokines. For example, administration of *Lactobacillus plantarum* NA136 reduces levels of TNF-α, IL-6, and IL-1β, thereby alleviating inflammation in the colon mucosa (42). Similarly, in our study, we showed that TS2 supplementation reduced the levels of IL-1β, IL-6 and TNF-α, suggesting that TS2 isolated from yaks exerted protective and beneficial effects in broilers by controlling cytokine secretion. Low-dose TS2 may regulate the balance of immune responses and inhibit the occurrence of excessive inflammatory reactions through its own actions or interactions with intestinal mucosa and immune cells, potentially stimulated by other probiotics.

During the rearing process, broilers may encounter various stressors, including environmental factors, transportation and handling, diseases, infections, and feed. In our previous experiments, we found that TS2 can produce antibacterial substances, such as antibacterial peptides, to inhibit the growth of harmful bacteria (such as *Salmonella*, *E. coli*, etc.), thus reducing the production of toxins and inflammatory mediators. In this study, we further discovered that low-dose TS2 can effectively reduce the occurrence of inflammatory reactions during the rearing of broilers by promoting the proliferation of beneficial bacteria, regulating immune function, and reducing oxidative stress.

Finally, it should be noted that individual differences and experimental conditions prohibited elimination of all factors that may influence the results of our study. Nevertheless, our data provide evidence that TS2 isolated from yaks can increase the growth performance of broilers by improving the length of intestinal villi. More importantly, broilers treated with low-dose TS2 exhibited superior properties in terms of antioxidant capacity and a reduction in the levels of cytokines related to immunity and inflammation. In conclusion, our study suggests that TS2 isolated from yaks can serve as a safe and effective probiotic additive, promoting the health and growth of the host.

## Conclusion

Our study confirms the safety of low-dose TS2 and demonstrates its ability to enhance broiler growth, increasing ADWG while decreasing FCR. Additionally, supplementation with low-dose TS2 improves intestinal mucosal morphology and the ratio of villi to crypt cells. This improvement may be linked to the enhanced abundance and diversity of gut microbiota in broilers. Moreover, low-dose TS2 enhances the antioxidant capacity and mitigates inflammatory responses in broilers by increasing antioxidant enzyme activity and reducing serum pro-inflammatory factors such as IL-1β, IL-6, and TNF-α. Although the effects of other concentrations of TS2 groups were either insignificant or absent, opting for low-dose TS2 supplementation is the most suitable approach for enhancing growth, antioxidant capacity, and mitigating inflammatory responses in broilers. Additionally, because TS2 exhibits good production performance, it has the potential for industrial-scale production.

## Data availability statement

The original contributions presented in the study are publicly available. This data can be found at: https://www.ncbi.nlm.nih.gov/bioproject/; PRJNA1080183.

## Ethics statement

The animal study was approved by the Animal Welfare Committee of Nanjing Agricultural University. The study was conducted in accordance with the local legislation and institutional requirements.

## Author contributions

CG: Writing – original draft, Investigation, Formal analysis, Conceptualization. SL: Writing – original draft, Investigation, Formal analysis, Conceptualization. LD: Writing – review & editing, Resources, Conceptualization. ST: Writing – review & editing, Supervision, Project administration, Funding acquisition, Conceptualization.
